# Mycotoxins and Mycotoxin Producing Fungi in Pollen: Review

**DOI:** 10.3390/toxins11020064

**Published:** 2019-01-24

**Authors:** Aleksandar Ž. Kostić, Danijel D. Milinčić, Tanja S. Petrović, Vesna S. Krnjaja, Sladjana P. Stanojević, Miroljub B. Barać, Živoslav Lj. Tešić, Mirjana B. Pešić

**Affiliations:** 1 Chemistry and Biochemistry, Faculty of Agriculture, University of Belgrade, Nemanjina 6, 11080 Belgrade, Serbia; danijel.milincic@agrif.bg.ac.rs (D.D.M.); sladjas@agrif.bg.ac.rs (S.P.S.); baracm@agrif.bg.ac.rs (M.B.B.); mpesic@agrif.bg.ac.rs (M.B.P.); 2 Preservation and Fermentation, Faculty of Agriculture, University of Belgrade, Nemanjina 6, 11080 Belgrade, Serbia; tpetrovic@agrif.bg.ac.rs; 3 Institute for Animal Husbandry, Autoput 16, 11080 Belgrade, Serbia; vesnakrnjaja.izs@gmail.com; 4 Analytical Chemistry, Faculty of Chemistry, University of Belgrade, Studentski Trg 12-16, 11158 Belgrade, Serbia; ztesic@chem.bg.ac.rs

**Keywords:** pollen, fungi, mycotoxins, aflatoxins, ochratoxins, fumonisins, T-2 toxin, zearalenone, deoxynivalenol

## Abstract

Due to its divergent chemical composition and good nutritional properties, pollen is not only important as a potential food supplement but also as a good substrate for the development of different microorganisms. Among such microorganisms, toxigenic fungi are extremely dangerous as they can synthesize mycotoxins as a part of their metabolic pathways. Furthermore, favorable conditions that enable the synthesis of mycotoxins (adequate temperature, relative humidity, pH, and a_w_ values) are found frequently during pollen collection and/or production process. Internationally, several different mycotoxins have been identified in pollen samples, with a noted predominance of aflatoxins, ochratoxins, fumonisins, zearalenone, deoxynivalenol, and T-2 toxin. Mycotoxins are, generally speaking, extremely harmful for humans and other mammals. Current EU legislation contains guidelines on the permissible content of this group of compounds, but without information pertaining to the content of mycotoxins in pollen. Currently only aflatoxins have been researched and discussed in the literature in regard to proposed limits. Therefore, the aim of this review is to give information about the presence of different mycotoxins in pollen samples collected all around the world, to propose possible aflatoxin contamination pathways, and to emphasize the importance of a regular mycotoxicological analysis of pollen. Furthermore, a suggestion is made regarding the legal regulation of pollen as a food supplement and the proposed tolerable limits for other mycotoxins.

## 1. Introduction

Pollen grain, as a male gametophyte of flowering plants, is produced and released from anthers during pollination [1]. Two of the most important pollinators are insects (in the case of entomophilous plants it is, above all, the honey bee (*Apis mellifera* L.)) and, in the case of anemophilous plants, wind. Pollen is prime food for bees due to its amazing diversity of nutritionally important constituents-proteins, lipids, carbohydrates, vitamins, and minerals [2,3]. For the same reasons, floral or bee-collected pollen is potentially a good food supplement for human nutrition [4,5,6,7,8]. Because of great its sensitivity, pollen grain contains a significant quantity of secondary plant metabolites, as part of the plant’s defense mechanism, such as different phenolic compounds [9,10,11,12,13,14,15,16] or carotenoids [17,18] and possesses substantial antioxidant properties, which is important for its application as a food supplement [19,20]. Besides the nutritionally important and desirable components, pollen can contain some contaminants such as toxic elements [2,21,22,23]. Due to optimal water (moisture) content, water activity (a_w_), and pH-value, pollen often presents an ideal medium for the development of different microorganisms—bacteria, mold, and yeast. As a result of the presence of mold and yeast, the production of mycotoxins can occur. Mycotoxins are secondary metabolites of different fungi species which are toxic to vertebrates and can lead to some disorders and diseases, or, at worst, death in humans and other animals [24]. The scientific “history” of mycotoxins started in 1962 during a great veterinary crisis when about 100,000 turkeys died in England due to being fed with contaminated peanuts that contained secondary metabolites of *Aspergillus flavus* [24]. The occurrence of mycotoxins in different types of feed and food has been recorded [25,26,27,28,29,30,31] and it was found to be strongly dependent on several factors such as climatic conditions (including geographical position of growing region, temperature, and relative humidity) before, during, or after feed/food production [32]. The European Commission (EC Commission Regulation No 1881/2006) sets maximum tolerable levels for several types of mycotoxins (aflatoxins B, G, and/or M, ochratoxin A (OTA), patulin, fumonisins B_1_ and B_2_, deoxynivalenol, and zearalenone) in different types of foods (nuts, cereals, dried fruits, juices, milk, etc.) [33] but without information pertaining to bee products such as honey, pollen, or bee bread.

The aim of this review is to make a cross-check of current data about contamination of pollen with different types of mycotoxins as well as mycotoxin producing fungi. Also, the effort to emphasize the importance of mycotoxin estimation of pollen samples as obligatory part of their microbiological analysis will be made.

## 2. Mycotoxins in Pollen

More than a hundred mycotoxins are known, and most of them are produced by some of the species belonging to one of three fungi genera: *Aspergillus*, *Penicillium* and/or *Fusarium* [34]. According to the available literature [35,36,37,38,39,40,41,42,43,44,45,46,47,48,49,50] the presence of the following mycotoxins in pollen has been investigated or proved with appropriate analytical methods and analysis: Aflatoxins (AFs), ochratoxins (OTs), fumonisins (FBs), zearalenone (ZEN), deoxynivalenol (DON), and its acetoxy derivative, T-2 toxin (T-2), HT-2 toxin, fusarenon-X, diacetoxyscirpenol, nivalenol, neosolaniol, roridin A, verrucarrin A, α-β-dehydrocurvularin, phomalactone,6-(1-propenyl)-3,4,5,6-tetrahydro-5-hydroxy-4H-pyran-2-one, 5-[1-(1hydroxibut-2-enyl)]-dihydrofuran-2-one and 5-[1-(1-hydroxibut-2-enyl)]-furan-2-one.

### 2.1. Aflatoxins

**Figure 1:**
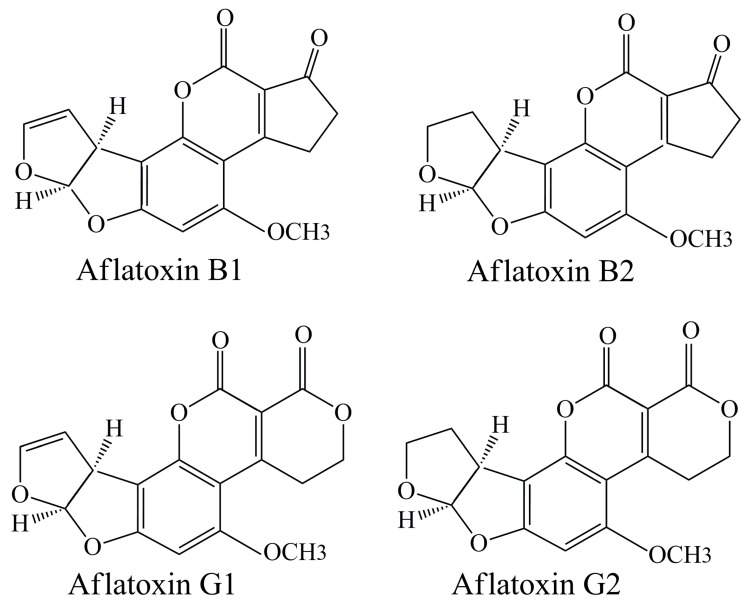
Chemical structures of aflatoxin B_1_, B_2_, G_1_, and G_2_.

Aflatoxins are the product of the metabolism of different fungi species which belong to *Aspergillus* genus with *A. flavus* and *A. parasiticus* strains as the main producers [24]. They can be synthetized in fungi’s spores and mycelium or secreted as exotoxins [25]. The most toxic and dangerous aflatoxins are aflatoxin B_1_ and B_2_ (Figure 1) [34]. Both aflatoxin B_1_ and B_2_ are carcinogenic for humans and animals, and are listed in Group 1 of carcinogenic substances according to International Agency for Research on Cancer (IARC) [51]. The liver is the organ that suffers most from the effects of aflatoxins [52]. Ingestion of these toxins can lead to aflatoxicosis, as an acute form of poisoning, or, in the case of long-term exposure, to the development of liver cancer [52]. Hydroxylated AFB-forms presented in milk are aflatoxin M1 and M2 [24] which are possibly carcinogenic for humans (IARC Group 2A of carcinogenic substances) [34,51]. Furthermore, two other forms of AF exist: Aflatoxin G1 and G2 (Figure 1).

#### 2.1.1. Contamination of Pollen with Aflatoxins—Possible Ways

Pollen often presents a suitable substrate for the proliferation of various microorganisms due to its favorable moisture content, water activity (a_w_), and pH-value. External conditions such as relative humidity and temperature, different stages of pollen production, and storage conditions have been shown to lead to microbiological contamination of pollen [35]. According to data found in the literature, pH-value ranging between 4.0 and 6.5 have been shown to be suitable for the development of bacteria, mold, and yeast while the minimal a_w_-values sufficient for the growth of *Aspergillus* and *Penicillium* spp. have been shown to be 0.71 to 0.96 [53] i.e., 0.55 in the case of pollen [54]. Microbiological contamination is strongly pH and temperature dependent and is also conditioned by the type of microorganism [53]. If proper conditions have been achieved in any phase of pollen production, the growth of microbes will occur which can cause aflatoxin production and the contamination of pollen. In addition to production process and human hygiene practices, which are the most important sources of aflatoxin contamination, sometimes microbe growth can be triggered by infected flowering plants [25,48]. Namely, during the flowering and the pollination process, *Aspergillus* spp. spores can germinate on female flower parts. Following this, the toxigenic fungal spores placed in the pollen tubes will grow and further infect the egg-cells [25]. If bees visit these flowers, the contaminated pollen grains will be transferred into the hives. Since there is intensive contact between bees when in the hive (due to highly organized bee societies) their “home” is the third possible source of aflatoxin pollen contamination [48]. As aflatoxins show detrimental effects on bee health, the incidence of these compounds in hives is undesirable. It is for this reason that the occurrence and production of propolis in hives is an effective way for bees to deal with AFs toxicity [55,56] which could indicate that this source of pollen contamination with aflatoxins is at least probable. In the past, aflatoxin occurrence in feed and food was a characteristic of tropic or sub-tropic regions due to favorable climatic conditions. Recently, with climatic changes, which extensively influences weather conditions in temperate areas (such as the majority of Europe), the presence of aflatoxins in these areas is becoming more frequent. The detection of aflatoxins in samples of pollen from the most diverse parts of the world (Table 1) is in accordance with this fact and is becoming a growing problem. Interestingly, in our previous investigation [48] the majority of examined pollen samples were sterile but all were contaminated with AFB_1_. This situation confirms three hypotheses:

- There are different ways of pollen contamination with aflatoxin(s).
- These toxins remain in samples with or without presence of appropriate fungi.
- It is extremely important to always perform mycotoxicological analysis together with microbiological characterization of pollen.

#### 2.1.2. Quantification of Aflatoxins in Pollen Samples

Results of different studies about the determination of aflatoxin content in pollen samples with diverse palynological (botanical) and geographical origins are given in Table 1.

### 2.2. Ochratoxins

**Figure 2:**
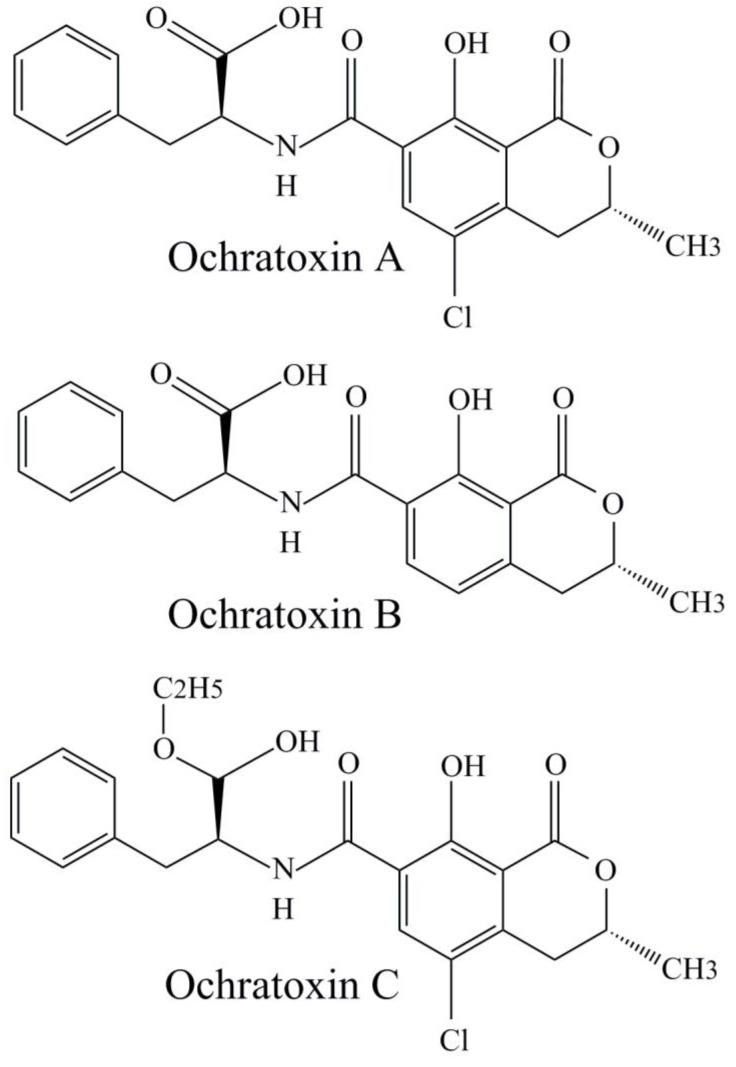
Chemical structures of ochratoxins A, B, and C.

Ochratoxins (OTs) are a group of chemical compounds (Figure 2) derived from shikimic acid metabolic pathway with ochratoxin A (OTA) as a major food contaminant [57]. The main OTs-producers are different *Aspergillus* species with a special emphasis on *Aspergillus niger* strains since they are industrially important due to their applications for enzyme and citric acid production. Furthermore, one species (*P. verrucosum*) belonging to *Penicillium* genus can be the source of ochratoxins [24]. OTA belongs to the IARC 2B group which means that it is a possible carcinogen for humans [51]. The kidneys are the most vulnerable organs effected by OTA. OTA has been noted as having a strong influence on the endemic disease ‘Balkan nephropathy’, as well as porcine nephropathy, which has been documented in several Scandinavian countries [24].

#### Ochratoxins in Pollen

Besides many types of food (nuts, meat products, barley, oats, rye, wheat, wine, dried fruits, coffee, and coffee products) where the presence of OTA has been recorded [24,57], in some herbs, bottled water [57], and pollen samples, this mycotoxin has also been observed. Xue et al. [45] conducted an examination of 20 bee pollen samples from North China for the presence of OTA p by LC-MS/MS analysis. The obtained results showed that none of the studied pollen samples were contaminated with OTA. These results can be associated with the dry weather conditions during the collection period. The same situation was observed in the case of 20 bee pollen samples that originated from Spain [37]. However, HPLC analysis of 90 Spanish and Argentinian bee pollen samples in [38] confirmed the presence of several *Aspergillus* (*A. carbonarius*, *A. ochraceus* and *A. niger*), and *Penicillium* (*P. verrucosum*) species with the ability to produce OTA. Significant contamination of bee pollen was determined in a case of Slovakian samples [41]. In total, 45 samples were divided in three groups of 15 samples originating from poppy, rape, and sunflower plants. Determined OTA concentration ranges in poppy, rape, and sunflower pollen samples were 6.12 to 10.98 μg/kg, 3.24 to 9.87 μg/kg, and 0.23 to 6.93 μg/kg, respectively. In Spain, by analyzing the toxigenic potential of *A. ochraceus* in various substrates (bee pollen, maize, wheat, and rice) Medina et al. [36] found that OTA production in bee pollen was statistically significantly higher than that found in the production of tested cereals, regardless of the incubation time (7, 14, 21, 28 days). Likewise, positive correlations have been found between the proportion of bee pollen added to the yeast extract sucrose broth inoculated with spores of *A. ochraceus* and OTA level [36]. Based on all of the above, it can be assumed that bee pollen may represent a significant risk factor for the occurrence of OTA in the food chain.

### 2.3. The Other Mycotoxins Examined in Pollen

#### 2.3.1. Fumonisins

**Figure 3:**
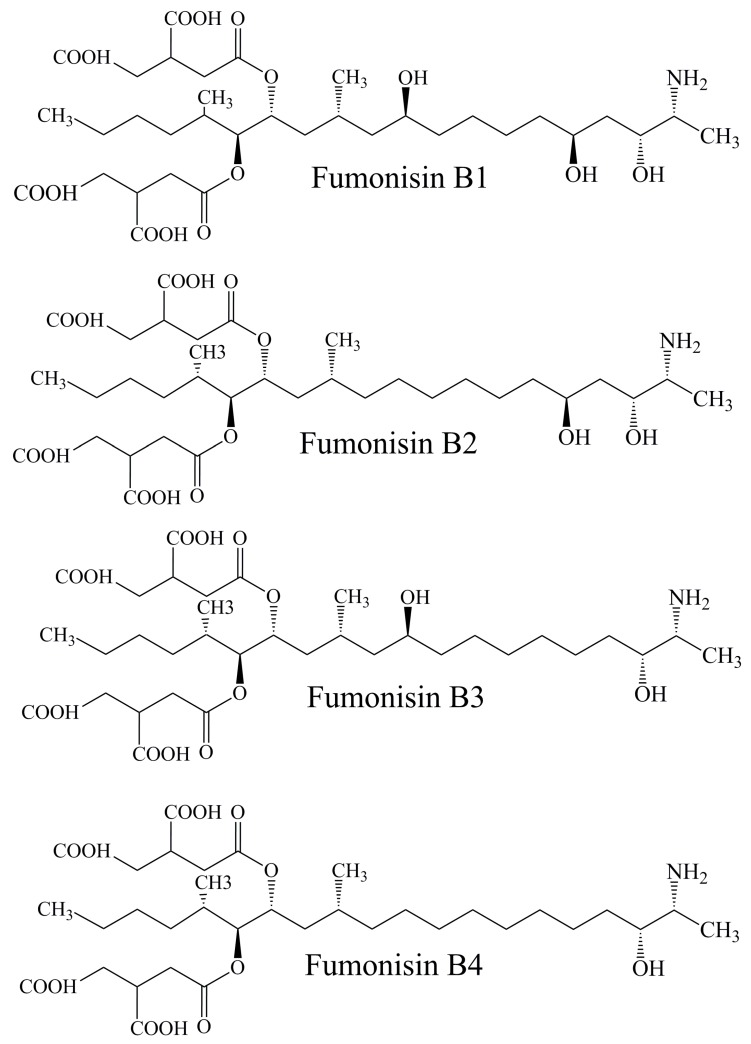
Chemical structures of fumonisins B_1_, B_2_, B_3_, and B_4_.

Fumonisins (FBs) are a group of mycotoxins predominantly connected with maize (grown as endophyte in both vegetative or reproductive tissues) and maize products but can be found in many cereals and products made from these plants [24,58]. Although maize is an anemophilic plant due to its high pollen production [7] it is not a rare that bees collect its pollen during the pollen collection season [4]. In that sense, it is possible to find pollen samples contaminated with FBs. The first report about FB food contamination dates back to 1988. The main representative of this mycotoxin group is fumonisin B_1_ (FB_1_) [24,58]. It is sorted in IARC 2B group of carcinogenic substances [51]. Moreover, fumonisins B_2_, B_3_, and B_4_ also exist (Figure 3) [57]. Fungi belonging to *Fusarium* genus are the most important FBs producers, especially two species: *F. proliferatum* and *F. verticillioides* as well as *A. alternata* from *Alternaria* spp. It is important to point out that the presence of these microbes does not mean that FBs contamination is guaranteed [24]. In an investigation by Kačaniová et al. [41] the presence of both, *F. proliferatum* and *F. verticillioides* was confirmed in thirty i.e., forty-five bee pollen samples, respectively but FBs were quantified only in the samples originating from sunflower (fifteen samples). This observation confirms the previously mentioned hypothesis, that despite the presence of *Fusarium* spp. in some material, appropriate weather conditions or insect damage are necessary for FBs production [24]. The range of FBs concentrations in these samples is given in Table 2.

#### 2.3.2. Zearalenone

**Figure 4:**
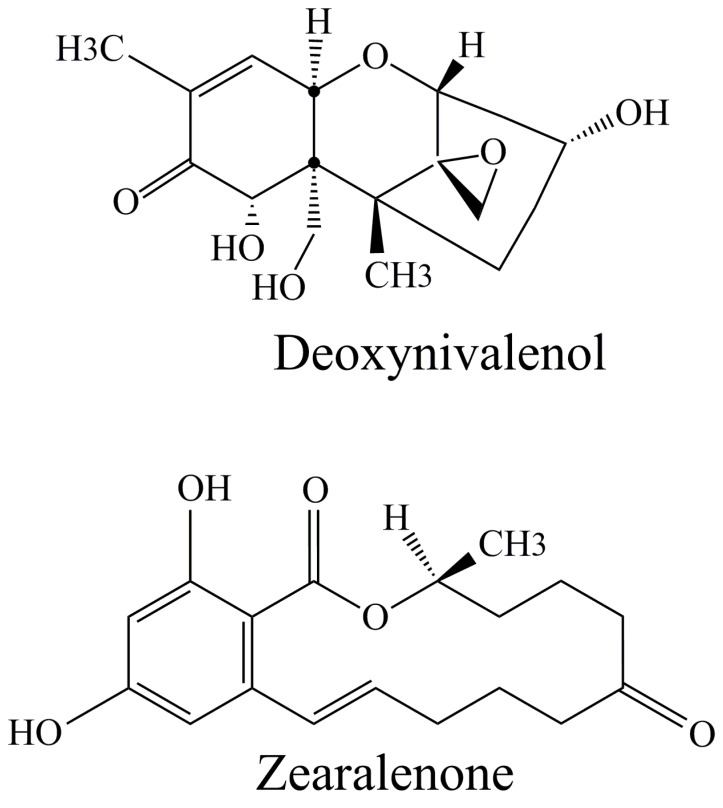
Chemical structures of deoxynivalenol and zearalenone.

Zearalenone (ZEN) (Figure 4) is mycoestrogen with limited toxicity that is produced by several *Fusarium* species: *F. graminearum*, *F. culmorum*, *F. crookwellense*, and *F. equiseti*. It is regularly present in crops and crop products [24]. According to IARC this macrocyclic lactone is classified in group 3 which means that it is not classifiable as to its carcinogenicity to humans [51]. In the case of pollen, the significant contamination with ZEN was recorded in Slovakian bee samples [41] (Table 2).

#### 2.3.3. Trichothecenes Group of Mycotoxins

In a study from Slovakia [41], the authors also reported the contamination of all examined bee pollen samples with T-2 toxin and deoxynivalenol (Figure 4). Both toxins belong to trichothecene compounds, the sesquiterpenoid metabolites obtained after microbiological activity of several fungi from the following genera: *Fusarium* (primary source), *Trichoderma*, *Myrothecium*, *Phomopsis*, etc., [24]. Together with ZEN, they were the most dominant quantified mycotoxins in the pollen samples. Additionally, the presence of DON and T-2 toxin was checked in fifteen pollen samples from Spain, but the content of these mycotoxins was below limit detection of applied GC/MS method [43]. In the same study, the authors examined the presence of several other *Fusarium* spp. producing mycotoxins: 3-acetyl-deoxynivalenol, fusarenon-X, diacetoxiscirpenol, nivalenol, neosolaniol, and HT-2 toxin. All the above-mentioned compounds belong to trichothecene terpenoid’s derivatives. It was determined that some of the samples were contaminated with neosolaniol and nivalenol (Table 2), while all other examined toxins were below limit detection. A report made by Cirigiliano et al. [46] should also be mentioned as their study was the first to detect seven specific mycotoxins (roridin A, verrucarrin A, α-β-dehydrocurvularin, phomalactone,6-(1-propenyl)-3-,4,5,6-tetrahydro-5-hydroxy-4H-pirane-2-one, 5-[1-(1-hydroxibut-2-enyl)]-dihydrofuran-2-one and 5-[1-(1-hydroxibut-2-enyl)]-furan-2-one) in beehives from Argentina with pronounced antifungal effect. Roridin A, verrucarin A, and α-β-dehydrocurvularin were isolated from strains of fungi *Myrothecium verrucaria* while other mycotoxins were obtained as result of *Nigrospora sphaerica* strains activity. Their structures were confirmed by 1D and 2D-NMR spectroscopy.

## 3. Mycotoxin Producing Fungi in Pollen

The microbiological quality of pollen is equally important as its chemical composition due to its safety use. Although the examination of mycotoxins in pollen began mostly in the last decade, the determination of different microbes (bacteria, mold, and yeast) present in pollen samples started much earlier—at the end of 1970s with studies by Gilliam [59,60]. Considering that a long period of time usually passes between collection of pollen samples and its application as food supplement (or as medicament), there is a great chance for the development of some toxigenic fungi [41]. Their presence may indicate mycotoxin production in pollen with or without their quantification. In that sense, this review also gives information on pollen investigations concerning the presence of mycotoxin producing fungi [41] made without further mycotoxicological analysis. The results of a cross-check of the available literature data, with appropriate comments and information, are given in Table 3.

## 4. Legislations of Mycotoxins Level in Food and Pollen

In order to prevent undesirable consequences and to protect consumers health, the European Commission, as well as some other international agencies, have proposed maximum permissible concentrations (MPC) for several mycotoxins in different types of food [33,81]. Maximum permissible concentrations vary due to differences in food origin and greater/less possibility of contamination with mycotoxins, as well as because of smaller or larger intake in meals. For instance, the MPC for AFB_1_ alters from 0 to 8 μg/kg [33]. Zero tolerance is established for milk and dairy products due to regular daily consumption while the maximal value has been proposed for groundnut-based food. Furthermore, for sensitive groups (such as infants and children), special lower limits have been usually established. The proposed limits are subject to corrections as a result of the development of new, more precise, and sensitive analytical methods for determining the content of mycotoxins [81]. In Table 4 current EU MPC values for some food types are given.

The Scientific Committee of Food requested and obtained from the European Food Safety Authority (EFSA) current data for Tolerable Weekly Intake (TWI) for OTA—0.12 μg/kg of body weight (bw) [82]. Recently, EFSA published new information about the potential increase of maximum allowable level (from 4 to 10 μg/kg) for total AFs in peanuts and processed products, requested by EU Commission [83]. The CONTAM panel (EFSA Panel on Contaminants in the Food Chain) strongly opposed this request due to the significant increase of cancer risk (factor value = 1.6–1.8). For other mycotoxins proposed Tolerable Daily Intake (TDI) values are: 2 μg/kg bw for nivalenol, 0.25 μg/kg bw for ZEN [84], 2 μg/kg (provisional maximum TDI) for FBs [85], 1 μg/kg bw for DON [86], 0.1 μg/kg bw for the sum of T-2 and HT-2 toxins [87], 0.06 μg/kg for combined trichothecenes mycotoxins group [33]. In these legislations, there is no information about proposed limits for mycotoxins in pollen. In 2008 Campos et al. [2] proposed that in the case of AFB_1_ occurrence in pollen the MPC value should be set at 2 μg/kg i.e., 4.2 μg/kg for total AFs. To the best of our knowledge, this is the only proposal which defines the level of some mycotoxins in pollen. Since this paper gives an overview about the presence of different mycotoxins in pollen samples originating from various locations around the world, it will be of great importance to define some tolerable levels for other fungi-produced toxins in pollen, especially for OTA. Moreover, current values for AFB_1_ and AFs should be reconsidered and checked due to an increasingly frequent aflatoxin contamination caused by climatic changes. Special concerns exist due to mixed (cross) contamination of pollen samples as confirmed by the presented data. Previously, several authors [32,88,89] confirmed that some combined mycotoxins have a more distinct detrimental effect on human health. Furthermore, Manafi et al. [90] have shown that AFs and T-2 toxin synergistically influenced the decrease of total serum protein and albumin levels in broiler chickens as well as decreased antibody titers. It is therefore of the utmost importance to evaluate the toxicological impact of mycotoxin combinations on animal and human health risks.

## 5. Conclusions and Future Perspectives

Pollen could be used as a food supplement which can be attributed to its appropriate chemical composition. The microbiological quality of pollen is equally important as its nutritional characteristics. The fungal contamination of different feed/food, including pollen will be more frequent as a result of intensive climatic changes. The quality of pollen can be significantly influenced by the presence of toxigenic fungi. Since it has been proved that the absence of microbial contamination in pollen does not exclude the presence of mycotoxins, mycotoxicological analyses should also be included as a regular control measure together with microbiological tests. Since aflatoxins and ochratoxins are proven as carcinogenic substances, their presence in pollen is extremely undesirable. Therefore, it is important to monitor mold and mycotoxin levels in feed/food in order to avoid adverse health effects. The incorporation of pollen as a food supplement in current legislation will be useful. Proposed quality parameters need to cover tolerable daily/weekly intake for different mycotoxins as well as their sum. In order to obtain reliable and accurate recommendations for pollen quality control, further studies on the toxicological impact of mycotoxin combinations should be conducted.

## Figures and Tables

**Table 1:** Toxigenic fungi and concentration level reported for aflatoxins in pollen samples from different countries.

No. of Examined Pollen Samples	Geographical Origin	Analytical Methods	Isolated Mycotoxins Producing Fungi Species	AF Types and Concentration Range(s)	Reference
20	Spain	ELISA test	/	Total AFs: below 5 μg/kg	[35]
20	Spain	HPLC (with fluorescent detection)	/	AFB_1_ and AFB_2_: below limit detection (BLD)	[37]
87 + 3	Spain + Argentina	HPLC (with fluorescent detection)	*A. flavus* *A. parasiticus*	AFB_1_, AFB_2_, AFG_1_ and AFG_2_: not determined.	[38]
5	China	Cyclic voltametry	/	AFB_1_: 0.00–0.52 μg/kg	[39,42]
1	Epirus (Western Greece)	HPLC (with fluorescent detection)	not detected	AFB_1_: not detected	[40]
45	Slovakia	ELISA test	*A. flavus*, *A. parasiticus*.	Total AFs: 13.60–16.20 μg/kg (in poppy pollen) 3.15–5.40 μg/kg (in rape pollen) 1.20–3.40 μg/kg (in sunflower pollen)	[41]
33	Serbia	ELISA test	*A. flavus*	AFB_1_: 3.49–14.02 μg/kg	[44]
20	China	LC-MS/MS	/	AFB_1_, AFB_2_, AFG_1_ and AFG_2_: below limit detection (BLD)	[45]
27	Brazil	Qualitative analysis	*A. flavus*	AFB_1_ and AFB_2_: not determined	[47]
26	Serbia	ELISA test	*A. flavus*	AFB_1_: 3.15–17.32 μg/kg	[48]
30	Egypt	Thin-layer chromatography	*A. flavus*	AFB_1_ AFB_2_, AFG_1_ and AFG_2_ were not determined.	[49]
9	Portugal	ELISA test	Not detected	Not detected AFB1	[50]

ELISA—enzyme linked immunosorbent assays; AFs—aflatoxins; AFB_1_—aflatoxin B_1_; AFB_2_—aflatoxin B_2_; AFG_1_—aflatoxin G_1_; AFG_2_—aflatoxin G_2_.

**Table 2:** Concentration level reported for mycotoxins other than aflatoxins in pollen samples from different countries.

No. of Contaminated/Examined Pollen Samples	Geographical Origin	Analytical Methods	Isolated Mycotoxin Producing Fungi Specie(s)	Mycotoxin Types and Concentration Range(s)	Reference
15/45 were contaminated	Slovakia	ELISA test	*F. proliferatum*, *A. alternata* Keissl.	Total FBs: 6.30–12.60 μg/kg	[41]
45	Slovakia	ELISA test	*F. graminearum*	ZEN: 311.00–361.30 μg/kg (in poppy pollen) 137.10–181.60 μg/kg (in rape pollen) 115.60–147.40 μg/kg (in sunflower pollen)	[41]
45	Slovakia	ELISA test	*F. graminearum*, *F. oxysporum*, *F. proliferatum*, *F. sporotrichioides*, *F. verticillioides*	T-2 toxin: 113.90–299.60 μg/kg (in poppy pollen) 197.10-265.70 μg/kg (in rape pollen) 173.60–364.90 μg/kg (in sunflower pollen)	[41]
45	Slovakia	ELISA test	*F. graminearum*, *F. oxysporum*, *F. proliferatum*, *F. sporotrichioides*, *F. verticillioides*	DON: 183.10–273.90 μg/kg (in poppy pollen) 189.60–244.70 μg/kg (in rape pollen) 133.30–203.50 μg/kg (in sunflower pollen)	[41]
2/15	Spain	GC/MS	/	neosolaniol: 22 i.e., 30 μg/kg nivalenol: 1 μg/kg	[43]

ELISA—enzyme linked immunosorbent assays; FBs—fumonisins; ZEN—zearalenone; DON—deoxynivalenol.

**Table 3:** Toxigenic fungi and yeast reported in pollen samples from different countries.

No. of Examined Pollen Samples	Geographical Origin	Detected Microbial Class	Microbial Species or/and Total Microbial	Microbial Count	Observations	Reference
Unknown number of samples of floral and bee-collected almond pollen	unknown	Mold		No. of fungal isolates:	*Mucor* spp. was the dominant mold in floral pollen but not identified in bee-collected pollen. *Aureobasidium pullulans*, *P. corylophilum*, *P. crustosum* and *Rhizopus nigricans* were identified only in bee-collected pollen.	[61]
			*Alternaria* spp.	6		
			*Cladosporium* spp.	5		
			*Penicillium* spp.	5		
			*Aspergillus* spp.	3		
			*Mucor* spp.	19		
90 samples of bee pollen	Spain (87 samples) Argentina (3 samples)	Mold	*Aspergillus* section *Nigri*	1.4 × 10–2.3 × 10^2^ cfu/g	The results show the occurrence of different mold species in pollen samples. *Penicillium*, *Alternaria*, and *Aspergillus* spp. were present in 90%, 86.6%, and 80% of samples, respectively. Predominant *Aspergillus* species was *A. niger*. The species of the genus *Fusarium* were isolated in 53.3%.	[38]
			*A.flavus* +*A. parasiticus*	1.7 × 10–2.5 × 10 cfu/g		
			Other *Aspergillus* spp.	2 × 10 cfu/g		
			*P. verrucosum*	1.4 × 10^2^ cfu/g		
			Other *Penicillium* spp.	1.3 × 10^2^–4.3 × 10^3^ cfu/g		
			*Fusarium* spp.	16–9.5 × 10^1^ cfu/g		
			*Cladosporium* spp.	6 × 10–1.4 × 10^3^ cfu/g		
			*Alternaria* spp.	6 × 10–5.2 × 10^2^ cfu/g		
			*Rhizopus* spp.	2 × 10–9 × 10 cfu/g		
			*Mucor* spp.	8–2.2 × 10^2^ cfu/g		
			*Botrytis* spp.	8–3 × 10 cfu/g		
			*Epicoccum* spp.	5–10 cfu/g		
		Yeast	Not specified	3.6 × 10^2^–7.3 × 10^3^ cfu/g		
42 samples of dehydrated bee pollen	Brazil	Mold/Yeast	Not specified	Total mold and yeast count: 10^2^–1.3 × 10^4^ cfu/g	About 12% of pollen samples were contaminated with mold and yeast above the limit (1×10^4^) for a total mold and yeast proposed by Brazilian legislation.	[62]
30 samples of bee pollen	Slovakia	Microscopic fungi (mold)	*Alternaria* spp. *Cladosporium* spp. *Penicillium* spp. *Fusarium* spp. *Aspergillus* spp. (*A. flavus*, *A. ochraceus*) *Mucor* spp. *Trichoderma* spp. *Acremonium* spp. *Scopulariopsis* spp. *Rhizopus* spp. *Botrytis* spp.	Total mold and yeast count: 1.1 × 10^2^–4.57 × 10^5^ cfu/g	The dominant fungi isolated from pollen samples were colonies of *A. alternata*, *Cladosporium cladosporoides*, and *Penicillium* spp. Also, the presence of well-known mycotoxicogenic species such as *A. flavus* and *A. ochraceus* were detected.	[63]
19 samples of bee pollen	Mexico	Fungi (mold)	*A. flavus*	Incidence of mold genus (%):	Fungi contamination was generally low. The highest contamination was in three samples handled without packages.	[64]
			*Alternaria* spp.	3.6%		
			*Penicillium* spp.	2.9%		
			*Fusarium* spp.	2.9%		
			*Aspergillus* spp.	3.6%		
			*Mucor* spp.	3.1%		
			*Rhizopus* spp.	0.7%		
8 samples of bee pollen	Slovakia	Mold	*Alternaria* spp. *Cladosporium* spp. *Penicillium* spp. *Aspergillus* spp. *Mucor* spp. *Aureobasidium* spp. *Humicola* spp. *Monodictys* spp. *Paecilomyces* spp. *Rhizopus* spp. *Mortierella* spp. *Trichosporiella* spp. *Harpografium* spp. *Mortierella* spp.	Total mold and yeast count: 107–4688 cfu/g	The results show that in all analyzed samples of pollen 21 fungal species of 13 genera of microscopic fungi were detected. The dominant identified species, over 62% of the isolates belonged to following genera: *Mucor, Rhizopus, Aspergillus, Alternaria,* and *Paecilomyces*.	[65]
28 samples (fresh and dried bee pollen)	Cuba	Mold/Yeast	Not specified	Total mold and yeast count: 10^4^–1.5 × 10^5^ cfu/g	All samples had quantified number of mold and yeast above proposed limits (10^4^ cfu/g for the fresh and 10^2^ cfu/g for dried pollen). Nevertheless, in the dry pollen, a smaller number of high contaminated samples were recorded. Drying could not be used as reliable method for obtaining pollen with acceptable microbiological quality.	[66]
8 samples of commercial bee pollen	Portugal (4 samples) Spain (3 samples) Unknown origin (1 sample)	Mold Yeast	Not specified Individually identified yeast	Total mold and yeast count: ˂10 to 9.4 × 10^2^ cfu/g	All samples were contaminated with yeast and mold. Further, yeast species were identified, and results indicated the presence of five different genus of yeast which can influence the risk of food-borne illness and spoilage or can serve as an indicator of a lack of hygiene standards.	[67]
Unknown	Portugal	Mold/Yeast	Not specified	Total mold and yeast count: ˂10^4^ cfu/g	Generally, yeast and mold were identified in 60% of all examined samples. pH and a_w_ values had a strong impact on the total microbe number in pollen.	[54]
22 samples of organic bee pollen	Portugal	Mold/Yeast	Not specified	Total mold and yeast count: ˂10–3560 cfu/g	In all samples of organic bee pollen, the presence of mold and yeast was detected, but their individual species were not identified.	[68]
3 samples of pollen	Algeria	Mold/Yeast	Not specified	Total mold and yeast count: 5 × 10^4^–4 × 10^5^ cfu/g	/	[69]
33 samples of bee pollen	Serbia	Mold	*Alternaria* spp. *Mucor* spp. *Rhizopus* spp. *Cladosporium* spp. *Epicoccum* spp. *Acremonium* spp.	Total mold count: 1 × 10^3^–1 × 10^5^ cfu/g	See Table 1.	[44]
27 samples of dried bee pollen	Brazil	Mold		Total mold count: 1 × 10^2^–5 × 10^2^ cfu/g Incidence of mold genus (%):	Total mold count depends on growing media.	[47]
			*Aspergillus* spp. (*A. flavus; A. fumigatus; A. versicolor; A. ochraceus; A. carbonarius; A. terreus; A. oryzae*)	85%		
			*Cladosporium* spp.	63%		
			*Penicillium* spp. (*P. citrinum; P. citreonigrum; P. glabrum; P. oxalicum*)	41%		
			*Alternaria* spp.	19%		
			*Wallemia* spp. and *Eurotium* spp.	11%		
			*Mucor* spp.	7%		
			*Curvularia* spp., *Paecilomyces* spp. and *Fusarium* spp. (*F. camptoceras*)	4%		
45 samples of dehydrated bee pollen	Brazil	Mold Yeast	Not specified Identified different species	Total mold and yeast count: ˂10–7.67 × 10^3^ cfu/g	/	[70]
21 samples of bee pollen (*Melipona* bees)	Brazil	Mold/Yeast	Not specified	/	All samples were sterile without presence of any mold or yeast species.	[71]
40 samples of bee pollen	Italy	Mold	*Cladosporium* spp. *Alternaria* spp. *Humicola* spp. *Mucoraceae* *Acremonium* spp. *Penicillium* spp. (*P. chrysogenum; P. brevicompacticum*) *Aspergillus* spp. (*A. flavus; A. nidulans; A.miger; A. terreus*)	Total mold count: 4–568 cfu/g	In all pollen samples at least one fungal isolate was detected. *Cladosporium* spp. was the most frequently detected mold. *Aspergillus* spp. and *Penicillium* spp., as a potentially mycotoxicogenic mold, were also identified in 8 i.e., 22 pollen samples.	[72]
Dehydrated (electric oven, EO) or lyophilized (L) bee pollen samples	Brazil	Mold/Yeast	Not specified	Total mold and yeast count: 99–242 cfu/g (EO) 16–935 cfu/g (L)	Number of quantified mold and yeast depended on time (April or September) of collection.	[73]
26 samples of bee pollen	Serbia	Mold		Total mold count:	See Table 1	[48]
			*Alternaria* spp.	1 × 10^3^ cfu/g		
			*Mucor* spp.	1 × 10^3^ cfu/g		
			*Rhizopus* spp.	1 × 10^3^ cfu/g		
			*Trichoderma* spp.	1 × 10^4^ cfu/g		
1 sample of bee pollen	Not known	Mold/Yeast	Not specified	Total mold and yeast count: >2l cfu/g	Presence of yeast and mold can be responsible for the potential presence of toxins in the samples.	[74]
18 samples of commercial bee pollen	Argentina	Mold/Yeast	Not specified	Total mold and yeast count: ˂10^2^ cfu/g	The total fungi number is specified for 28% of the samples.	[75]
62 samples of dehydrated bee pollen	Brazil	Mold/Yeast	Not specified	Total mold and yeast count: 1.9 × 10^2^–7.62 × 10^2^ cfu/g	The microbial contamination is dependent on geographical origin of samples.	[76]
8 samples of commercial bee pollen	Algeria	Mold/Yeast	Not specified	Total mold and yeast count: 10^4^–2.8 × 10^5^ cfu/g	/	[77]
32 (13 fresh (F) and 19 dried (D) samples of bee pollen)	Bulgaria	Mold	Identified mold: *Aspergillus* spp. *Fusarium* spp. *Penicillium* spp. (*P. brevicompactum*) *Alternaria* spp. *Cladosporium* spp. Other species	Total mold count: 5.6 × 10^2^ –3.7 × 10^4^ cfu/g (F) 150–1.1 × 10^4^ cfu/g (D)	The results show that the values for fungal colony count were significantly lower in the dried pollen samples. 136 fungal isolates were identified. Among detected isolates, genus *Penicillium* was dominant while the genus *Fusarium* was the least fungal contaminant. Dominant species isolated from 14 different samples was *P. brevicompactum*.	[78]
19 samples of stored pollen of five stingless bee species	Brazil	Mold/Yeast	Not specified	Total mold and yeast count: 4.2 × 10^1^ cfu/g (1 sample only)	The results show that only for the stored pollen of the stingless bee specie *Frieseomellite varies* it was possible to enumerate mold and yeast.	[79]
bee pollen samples	Colombia	Mold/Yeast	Not specified	Total mold and yeast count: 3 × 10^2^–2 × 10^5^ cfu/g	Number of quantified microbes is strongly dependent on applied temperature for drying of samples.	[80]

**Table 4:** Examples for the current maximum permissible concentrations (MPC) for some mycotoxins in different types of food/food supplements.

Food/Food Supplements	Mycotoxin(s)	MPC Value(s)	Reference
Groundnuts used as components for food production	AFB_1_	8 μg/kg	[33]
	Sum of AFB_1_, AFB_2_, AFG_1_ and AFG_2_	15 μg/kg	
Groundnuts for direct human consumption	AFB_1_	2 μg/kg	[33]
	Sum of AFB_1_, AFB_2_, AFG_1_ and AFG_2_	4 μg/kg	
Dried fruits used as components for food production	AFB_1_	5 μg/kg	[33]
	Sum of AFB_1_, AFB_2_, AFG_1_ and AFG_2_	10 μg/kg	
Dried fruits for direct human consumption	AFB_1_	2 μg/kg	[33]
	Sum of AFB_1_, AFB_2_, AFG_1_ and AFG_2_	4 μg/kg	
Raw milk used for consumption and dairy productions, infant formulae and infant-milk	AFB_1_	0 μg/kg	[33]
	Sum of AFB_1_, AFB_2_, AFG_1_ and AFG_2_	0 μg/kg	
Unprocessed cereals	OTA	5 μg/kg	[33]
Cereals based products	OTA	3 μg/kg	[33]
Instant coffee	OTA	10 μg/kg	[33]
Roasted coffee	OTA	5 μg/kg	[33]

## References

[1] Borg M., Brownfield L., Twell D. (2009). Male gametophyte development: A molecular perspective. J. Exp. Bot..

[2] Campos G.R.M., Bogdanov S., Almeida-Muradian L.B., Szczesna T., Mancebo Y., Frigerio C., Ferreira F. (2008). Pollen composition and standardization of analytical methods. J. Apic. Res..

[3] Bogdanov S. (2012). Pollen: Collection, harvest, composition, quality. Bee Product Science (The Pollen Book).

[4] Kostić A.Ž., Barać M.B., Stanojević S.P., Milojković-Opsenica D.M., Tešić Ž.L., Šikoparija B., Radišić P., Prentović M., Pešić M.B. (2015). Physicochemical properties and techno-functional properties of bee pollen collected in Serbia. LWT Food Sci. Technol..

[5] Kostić A.Ž., Kaluđerović L.M., Dojčinović B.P., Barać M.B., Babić V.B., Mačukanović-Jocić M.P. (2017). Preliminary investigation of mineral content of pollen collected from different Serbian maize hybrids—Is there any potential nutritional value?. J. Sci. Food Agric..

[6] Kostić A.Ž., Pešić M.B., Trbović D., Petronijević R., Dramićanin A., Milojković-Opsenica D.M., Tešić Ž.L. (2017). Fatty acid’s profile of Serbian bee-collected pollen—Chemotaxonomic and nutritional approach. J. Apic. Res..

[7] Kostić A.Ž., Mačukanović-Jocić M.P., Špirović Trifunović B.D., Vukašinović I.Ž., Pavlović V.B., Pešić M.B. (2017). Fatty acids of maize pollen-quantification, nutritional and morphological evaluation. J. Cereal Sci..

[8] Conte P., Del Caro A., Balestra F., Piga A., Fadda C. (2018). Bee pollen as a functional ingredient in gluten-free bread: A physical-chemical, technological and sensory approach. LWT Food Sci. Technol..

[9] Campos M., Markham M.R., Mitchell K.A., da Cuhna A.P. (1997). An approach to the characterization of bee pollens via their flavonoid/phenolic profiles. Phytochem Anal..

[10] Serra-Bonvehí J., Torrentó S.M., Lorente C.E. (2001). Evaluation of polyphenolic and flavonoid compounds in honeybee-collected pollen produced in Spain. J. Agric. Food Chem..

[11] Campos M.G., Webby F.B., Markham M.R., Mitchell K.A., da Cuhna A.P. (2003). Age-induced diminution of free radical scavenging capacity in bee pollens and the contribution of constituent flavonoids. J. Agric. Food Chem..

[12] Di Paola-Naranjo R.D., Sánchez S.J., Paramás A.M.G., Gonzalo J.C.R. (2004). Liquid chromatographic-mass spectrometric analysis of anthocyanin composition of dark blue bee pollen from *Echium Plantagineum*. J. Chromatogr. A.

[13] Almaraz Abarca N., Campos da Graça M., Ávila-Reyes J.A., Naranjo-Jiménez N., Corral J.H., González-Valdez L.S. (2007). Antioxidant activity of polyphenolic extract of monofloral honeybee-collected pollen from mesquite (*Prosopis juliflora, Leguminosae*). J. Food Compos. Anal..

[14] Ferreres F., Pereira D.M., Valentão P., Andrade P.B. (2010). First report of noncoloured flavonoids in *Echium plantagineum* bee pollen: Differentation of ismomers by liquid chromatography/ion trap mass spectometry. Rapid Commun. Mass Spectrom..

[15] Ares A.M., Valverde S., Bernal J.L., Nozal M.J., Bernal J. (2018). Extraction and determination of bioactive compounds from bee pollen. J. Pharm. Biomed. Anal..

[16] De-Melo A.A.M., Estevinho L.M., Moreira M.M., Delerue-Matos C., da Silva de Freitas A., Barth O.M., de Almeida-Muradian L.B. (2018). Phenolic profile by HPLC-MS, biological potential, and nutritonal value of a promising food: Monofloral bee pollen. J. Food Biochem..

[17] Almeida-Muradian L.B., Pamplona L.C., Coimbra S., Ortrud M.B. (2005). Chemical composition and botanical evaluation of dried bee pollen pellets. J. Food Compos. Anal..

[18] Mărgăoan R., Mărghitaş L.A., Dezmirean D.S., Dulf F.V., Bunea A., Socaci S.A., Bobiş O. (2014). Predominant and secondary pollen botanical origins influence the carotenoid and fatty acid profile in fresh honeybee-collected pollen. J. Agric. Food Chem..

[19] Krystyjan M., Gumul D., Ziobro R., Korus A. (2015). The fortification of biscuits with bee pollen and its effect on physicochemical and antioxidant properties in biscuits. LWT Food Sci. Technol..

[20] De Florio Almeida J., Soares dos Reis A., Serafini Heldt L.F., Pereira D., Bianchin M., de Moura C., Plata-Oviedo M.V., Haminiuk C.W.I., Ribeiro I.S., Fernades Pinto da Luz C. (2017). Lyophilized bee pollen extract: A natural antioxidant source to prevent lipid oxidation in refrigerated sausages. LWT Food Sci. Technol..

[21] Kostić A.Ž., Pešić M.B., Mosić M.D., Dojčinović B.P., Natić M.N., Trifković J.Đ. (2015). Mineral content of some bee-collected pollen from Serbia. Arch. Ind. Hyg. Toxicol..

[22] Sattler J.A.G., de Melo Machado A.A., do Nascimento K.S., de Melo Pereira I.L., Mancini-Filho J., Sattler A., de Almeida-Muradian L.B. (2016). Essential minerals and inorganic contaminants (barium, cadmium, lithium, lead and vanadium) in dried bee pollen produced in Rio Grande do Sul State, Brazil. Food Sci. Technol..

[23] Altunaltmaz S.S., Tarhan D., Aksu F., Barutçu U.B., Or M.E. (2017). Mineral element and heavy metal (cadmium, lead and arsenic) levels of bee pollen in Turkey. Food Sci. Technol..

[24] Bennet J.W., Klich M. (2003). Mycotoxins. Clin. Microbiol. Rev..

[25] Hanssen E., Jung M. (1973). Control of aflatoxins in the food industry. Pure Appl. Chem..

[26] Bosco F., Mollea C., Valdez B. (2012). Mycotoxins in food. Food Industrial Processes—Methods and Equipment.

[27] Stanković S., Lević J., Ivanović D., Krnjaja V., Stanković G., Tančić S. (2012). Fumonisin B1 and its co-occurrence with other fusariotoxins in naturally-contaminated wheat grain. Food Control.

[28] Krnjaja V., Mandić V., Lević J., Stanković S., Petrović T., Vasić T., Obradović A. (2015). Influence of N-fertilization on Fusarium head blight and mycotoxin levels in winter wheat. Crop Prot..

[29] Abrunhosa L., Morales H., Soares C., Calado T., Vila-Cha A.S., Pereira M., Venâncio A. (2016). A review of mycotoxins in food and feed products in Portugal and estimation of probable daily intake. Crit. Rev. Food Sci. Nutr..

[30] Bijelić Z., Krnjaja V., Stanković S., Muslić-Ružić D., Mandić V., Škrbić Z., Lukić M. (2017). Occurrence of moulds and mycotoxins in grass-legume silages influenced by nitrogen fertilization and phenological phase at harvest. Rom. Biotech. Lett..

[31] Krnjaja V., Stanković S., Obradović A., Petrović T., Mandić V., Bijelić Z., Božić M. (2018). Trichothecene genotypes of *Fusarium graminearum* populations isolated from winter wheat crops in Serbia. Toxins.

[32] Smith M.-C., Madec S., Coton E., Hymery N. (2016). Natural co-occurrence of mycotoxins in foods and feeds and their in vitro combined toxicological effects. Toxins.

[33] EC Commission (2006). Setting of maximum levels for certain contaminants in foodstuffs—Regulation No. 1881/2006. Official J. of the EU..

[34] Van Egmond H.P. (2013). Mycotoxins: Risks, regulations and European co-operation. J. Nat. Sci. Matica Srpska Novi Sad..

[35] Serra-Bonvehi J., Escolà Jordà R. (1997). Nutrient composition and microbiological quality of honey bee-collected pollen in Spain. J. Agric. Food Chem..

[36] Medina Á., González G., Sáez J.M., Mateo R., Jiménez M. (2004). Bee pollen, a substrate that stimulates ochratoxin A production by *Aspergillus ochraceus* Wilh. Syst. Appl. Microbiol..

[37] Garcia-Villanova R.J., Cordón C., González-Paramás A.M., Aparicio P., Garcia Rosales M.E. (2004). Simultaneous immunoaffinity column cleanup and hplc analysis of aflatoxins and ochratoxin a in spanish bee pollen. J. Agric. Food Chem..

[38] González G., Hinojo M.J., Mateo R., Medina A., Jiménez M. (2005). Occurrence of mycotoxin producing fungi in bee pollen. Int. J. Food Microbiol..

[39] Zaijun L., Zhongyun W., Xiulan S., Yinjun F., Peipei C. (2010). A sensitive and highly stable electrochemical impendace immunosensor based on the formation of silica gel-ionic liquid biocompatible film on the glassy carbon electrode for the determination of aflatoxin B1 in bee pollen. Talanta.

[40] Pitta M., Markaki P. (2010). Study of aflatoxin B1 production by *Aspergillus parasiticus* in bee pollen of Greek origin. Mycotoxin Res..

[41] Kačaniová M., Juráček M., Chlebo R., Kňazovická V., Kadasi-Horáková M., Kunová S., Lejková J., Haščik P., Mareček J., Šimko M. (2011). Mycobiota and mycotoxins in bee pollen collected from different areas of Slovakia. J. Environ. Sci. Health Part B.

[42] Vidal J.C., Bonel L., Ezquerra A., Hernández S., Bertolín J.R., Cubel C., Castillo J.R. (2013). Electrochemical affinity biosensors for detection of mycotoxins: A review. Biosens. Bioelectron..

[43] Rodríguez-Carasco Y., Font G., Mañes J., Berrada H. (2013). Determination of mycotoxins in bee pollen by gas chromatography−tandem mass spectrometry. J. Agric. Food Chem..

[44] Petrović T., Nedić N., Paunović D., Rajić J., Matović K., Radulović Z., Krnjaja V. (2014). Natural mycobiota and aflatoxin B1 presence in bee pollen collected in Serbia. Biotechnol. Anim. Husb..

[45] Xue X., Selvaraj J.N., Zhao L., Dong H., Liu F., Liu Y., Li Y. (2014). Simultaneous determination of aflatoxins and ochratoxin a in bee pollen by low-temperature fat precipitation and immunoaffinity column cleanup coupled with LC-MS/MS. Food Anal. Methods.

[46] Cirigliano A.M., Rodríguez M.A., Godeas A.M., Cabrera G.M. (2014). Mycotoxins from beehive pollen mycoflora. J. Sci. Res. Rep..

[47] Valadares Deveza M., Keller K.M., Affonso Lorenzon M.C., Teixeira Nunes L.M., Oliveira Sales E., Barth O.M. (2015). Mycotoxicological and palynological profiles of commercial brands of dried bee pollen. Braz. J. Microbiol..

[48] Kostić A.Ž., Petrović T.S., Krnjaja V.S., Nedić N.M., Tešić Ž.L., Milojković-Opsenica D.M., Barać M.B., Stanojević S.P., Pešić M.B. (2017). Mold/aflatoxin contamination of honey bee collected pollen from different Serbian regions. J. Apic. Res..

[49] Hosny A.S., Sabbah F.M., El-Bazza Z.E. (2018). Studies on microbial decontamination of Egyptian bee pollen by γ-irradiation. Egypt Pharm. J..

[50] Estevinho L.M., Dias T., Anjos O. (2018). Influence of the storage conditions (frozen vs dried) in health-related lipid indexes and antioxidants of bee pollen. Eur. J. Lipid Sci. Technol..

[51] Vidal A., Mengelers M., Yang S., De Saeger S., De Boevre M. (2018). Mycotoxin biomarkers of exposure: A comprehensive review. Compr. Rev. Food Sci. Food Saf..

[52] Neal G.E. (1995). Genetic implications in the metabolism and toxicity of mycotoxins. Toxicol. Lett..

[53] Magan N., Lacey J. (1984). Effect of temperature and pH on water realtions of field and storage fungi. Trans. Br. Mycol. Soc..

[54] Estevinho L.M., Rodrigues S., Pereira A.P., Feás X. (2012). Portugese bee pollen: Palynological study, nutritional and microbiological evaluation. Int. J. Food Sci. Technol..

[55] Niu G., Johnson R.M., Berenbaum M.R. (2011). Toxicity of mycotoxins to honeybees and its amelioration by propolis. Apidologie.

[56] Temiz A., Şener Mumcu A., Özkök Tüylü A., Sorkun K., Salih B. (2013). Antifungal activity of propolis samples collected from different geographical regions of Turkey against two food-related molds, *Aspergillus versicolor* and *Penicillium aurantiogriseum*. Gida.

[57] Tao Y., Xie S., Xu F., Liu A., Wang Y., Chen D., Pan Y., Huang L., Peng D., Wang X. (2018). Ochratoxin A: Toxicity, oxidative stress and metabolism (Review). Food Chem. Toxicol..

[58] Cendoya E., Chiotta M.L., Zachetti V., Chulze S.N., Ramirez M.L. (2018). Fumonisins and fumonisin-producing *Fusarium* occurrence in wheat and wheat by products: A review. J. Cereal Sci..

[59] Gilliam M. (1979). Microbiology of pollen and bee bread: The yeasts. Apidologie.

[60] Gilliam M. (1979). Microbiology of pollen and bee bread: The genus *Bacillus*. Apidologie.

[61] Gilliam M., Prest D.B., Lorenz B.J. (1989). Microbiology of pollen and bee bread: Taxonomy and enzimology of molds. Apidologie.

[62] Carelli Barreto L.M.R., Cunha Funari S.R., de Oliveira Rosi R. (2005). Composição e qualidade do pólen apícola proveniente de sete estados Brasileiros e do distrito federal. Bol. Ind. Anim..

[63] Kačániová M., Pavličová S., Haščík P., Kociubinski G., Kńazovická V., Sudzina M., Sudzinova J., Fikselová M. (2009). Microbial communities in bees, pollen and honey from Slovakia. Acta Microbiol. Immunol. Hung..

[64] Bucio Villalobos C.M., López Preciado G., Martínez Jaime O.A., Torres Morales J.J. (2010). Micoflora asociada a granos de polen recolectados por abejas domésticas (*Apis mellifera* L.). Rev. Electron. Nova Sci..

[65] Brindza J., Gróf J., Bacigálová K., Ferianc P., Tóth D. (2010). Pollen microbial colonization and food safety. Acta Chim. Slov..

[66] Puig-Peña Y., del-Risco-Ríos C.A., Álvarez-Rivera V.P., Leiva-Castillo V., García-Neninger R. (2012). Comparación de la calidad microbiológica del polen apícola fresco y después de un proceso de secado. Rev. CENIC. Cienc. Biol..

[67] Nogueira C., Iglesias A., Feás X., Estevinho M.L. (2012). Commercial bee pollen with different geographical origins: A comprehensive approach. Int. J. Mol. Sci..

[68] Feás X., Pilar Vázquez-Tato M., Estevinho L., Seijas J.A., Iglesias A. (2012). Organic bee pollen: Botanical origin, nutritional value, bioactive compounds, antioxidant activity and microbiological quality. Molecules.

[69] Hani B., Dalila B., Saliha D., Daoud H., Mouloud G., Seddik K. (2012). Microbiological sanitary aspects of pollen. Adv. Environ. Biol..

[70] De-Melo Machado A.A., Estevinho M.L.M.F., Almeida-Muradian L.B. (2015). A diagnosis of the microbiological quality of dehydrated bee-pollen produced in Brazil. Lett. Appl. Microbiol..

[71] Santa Bárbara M., Machado C.S., da Silva Sodré G., Dias L.G., Estevinho L.M., Lopes de Carvalho C.A. (2015). Microbiological assessment, nutritional characterization and phenolic compounds of bee pollen from *Mellipona mandacaia* Smith, 1983. Molecules.

[72] Nardoni S., D’Ascenzi C., Rocchigiani G., Moretti V., Mancianti F. (2016). Occurrence of molds from bee pollen in Central Italy—A preliminary study. Ann. Agric. Environ. Med..

[73] De-Melo Machado A.A., Fernandes Estevinho M.L.M., Gasparotto Sattler J.A., Rodrigues Souza B., da Silva Freitas A., Barth O.M., Bicudo Almeida-Muradian L. (2016). Effect of processing conditions on characteristics of dehydrated bee-pollen and correlation between quality parameters. LWT Food Sci. Technol..

[74] Grabowski N.T., Klein G. (2017). Microbiology of processed edible insect products—Results of a preliminary survey. Int. J. Food Microbiol..

[75] Libonatti C., Andersen-Puchuri L., Tabera A., Varela S., Passucci J., Basualdo M. (2017). Caracterización microbiológica de polen comercial. Reporte preliminar. Rev. Electron. Vet..

[76] Aparecida Soares de Arruda V., Vieria dos Santos A., Figueiredo Sampaio D., da Silva Araújo E., de Castro Peixoto A.L., Fernandes Estevinho L.M., de Almeida-Muradian B.L. (2017). Microbiological quality and physicochemical characterization of Brazilian bee pollen. J. Apic. Res..

[77] Adjlane N., Hadj Ali L.M., Benamara M., Bounadi O., Haddad N. (2017). Qualite microbiologique du pollen produit par les apiculteurs et commercialise en Algerie. Rev. Microbiol. Ind. San et Environ..

[78] Beev G., Stratev D., Vashin I., Pavlov D., Dinkov D. (2018). Quality assessment of bee pollen: A cross sectional survey in Bulgaria. J. Food Qual. Hazards Control.

[79] Figueredo Santa Bárbara M., Santiago Machado C., da Silva Sodré G., de Lima Silva F., Alfredo Lopes de Carvalho C. (2018). Microbiological and physicochemical characterization of the pollen stored by stingless bees. Braz. J. Food Technol..

[80] Zuluaga-Domínguez C., Serrato-Bermudez J., Quicazán M. (2018). Influence of drying-related operations on microbiological, structural and physicochemical aspects for processing of bee-pollen. Eng. Agric. Environ. Food..

[81] Arroyo-Manzanares N., Huertas-Pérez J.F., García-Campaña A.M., Gámiz-Gracia L. (2014). Mycotoxin analysis: New proposals for sample treatment. Adv. Chem..

[82] European Food Safety Authority (EFSA) (2006). Opinion of the Scientific Panel on contaminants in the food chain of the EFSA on a request from the Commission related to ochratoxin A in food. EFSA J..

[83] Knutsen H.K., Barregard L.J.A., Bingami M., Bruschweiler B., Ceccatelli S., Cottrill B., Dinovi M., Edler L., Grasl-Kraup B., EPSA Panel on Contaminants in the Food Chain (Contam) (2018). Effect on public health of a possible increase of the maximum level for ‘aflatoxin total’ from 4 to 10 μg/kg in peanuts and processed products thereof, intended for direct human consumption or use as an ingredient in foodstuffs-statement. EFSA J..

[84] EPSA Panel on Contaminants in the Food Chain (Contam) (2011). Scientific Opinion on the risks for public health related to the presence of zearalenone in food. EFSA J..

[85] Knutsen H.K., Alexander J., Barregard L.J.A., Bingami M., Bruschweiler B., Ceccatelli S., Cottrill B., Dinovi M., Grasl-Kraup B., EPSA Panel on Contaminants in the Food Chain (Contam) (2014). Scientific Opinion on the risks for human and animal health related to the presence of modified forms of certain mycotoxins in food and feed. EFSA J..

[86] EPSA Panel on Contaminants in the Food Chain (Contam) (2017). Risks to human and animal health related to the presence of deoxynivalenol and its acetylated and modfied forms in food and feed. EFSA J..

[87] EPSA Panel on Contaminants in the Food Chain (Contam) (2011). Scientific Opinion on the risks for animal and public health related to the presence of T-2 and HT-2 toxin in food and feed. EFSA J..

[88] Šegvić Klarić M. (2012). Adverse effects of combined mycotoxins. Arh. Ind. Hyg. Toxikol..

[89] Šegvić Klarić M., Rašić D., Peraica M. (2013). Deleterious effects of mycotoxin combinations involving Ochratoxin, A. Toxins.

[90] Manafi M., Umakantha B., Mohan K., Narayana Swamy H.D. (2012). Synergistic effects of two commonly contaminating mycotoxins (Aflatoxin and T-2 toxin) on biochemical parameters and immune status of broiler chickens. World Appl. Sci. J..

